# Inducible biosynthesis and immune function of the systemic acquired resistance inducer *N*-hydroxypipecolic acid in monocotyledonous and dicotyledonous plants

**DOI:** 10.1093/jxb/eraa317

**Published:** 2020-07-06

**Authors:** Anika Schnake, Michael Hartmann, Stefan Schreiber, Jana Malik, Lisa Brahmann, Ipek Yildiz, Janina von Dahlen, Laura E Rose, Ulrich Schaffrath, Jürgen Zeier

**Affiliations:** 1 Institute for Molecular Ecophysiology of Plants, Department of Biology, Heinrich Heine University, Universitätsstraße 1, Düsseldorf, Germany; 2 Institute for Population Genetics, Department of Biology, Heinrich Heine University, Universitätsstraße 1, Düsseldorf, Germany; 3 Department of Plant Physiology, RWTH Aachen University, Aachen, Germany; 4 Cluster of Excellence on Plant Sciences (CEPLAS), Heinrich Heine University, Universitätsstraße 1, Düsseldorf, Germany; 5 University of Edinburgh, UK

**Keywords:** *Brachypodium*, cucumber, *Magnaporthe*, *N*-hydroxypipecolic acid, pipecolic acid, plant immunity, *Pseudomonas*, salicylic acid, systemic acquired resistance, tobacco

## Abstract

Recent work has provided evidence for the occurrence of *N*-hydroxypipecolic acid (NHP) in *Arabidopsis thaliana*, characterized its pathogen-inducible biosynthesis by a three-step metabolic sequence from l-lysine, and established a central role for NHP in the regulation of systemic acquired resistance. Here, we show that NHP is biosynthesized in several other plant species in response to microbial attack, generally together with its direct metabolic precursor pipecolic acid and the phenolic immune signal salicylic acid. For example, NHP accumulates locally in inoculated leaves and systemically in distant leaves of cucumber in response to *Pseudomonas syringae* attack, in *Pseudomonas*-challenged tobacco and soybean leaves, in tomato inoculated with the oomycete *Phytophthora infestans*, in leaves of the monocot *Brachypodium distachyon* infected with bacterial (*Xanthomonas translucens*) and fungal (*Magnaporthe oryzae*) pathogens, and in *M. oryzae*-inoculated barley. Notably, resistance assays indicate that NHP acts as a potent inducer of acquired resistance to bacterial and fungal infection in distinct monocotyledonous and dicotyledonous species. Pronounced systemic accumulation of NHP in leaf phloem sap of locally inoculated cucumber supports a function for NHP as a phloem-mobile immune signal. Our study thus generalizes the existence and function of an NHP resistance pathway in plant systemic acquired resistance.

## Teaser

*N*-hydroxypipecolic acid, which systemically accumulates in phloem sap of *Pseudomonas syringae*-inoculated cucumber, is synthesized and functions as an activator of systemic acquired resistance in diverse monocotyledonous and dicotyledonous plant species.

## Introduction

Plant metabolites play diverse basic roles in plant defense, since they can act as antimicrobial weapons to directly harm attacking pathogens, contribute to the fortification of plant cell walls to impede the entrance of pathogens into plant tissue, and coordinate the inducible expression of components of plant immune responses (Ahuja *et al.*, 2012; Berens *et al.*, 2017; Chezem *et al.*, 2017). Salicylic acid (SA) is a central metabolic activator of plant immunity that guarantees the effective induction of resistance responses in the defense of plants against bacterial, fungal, oomycete, and viral infections. In many species, the levels of SA and SA derivatives such as SA-β-glucoside (SAG) and SA glucose ester in the leaf tissue of unstressed plants are low but significantly increase in response to pathogen attack (Klessig *et al.*, 2018). In the dicot model *Arabidopsis thaliana*, genetic and biochemical evidence indicates that this stress-inducible biosynthesis of SA proceeds via conversion of the shikimate pathway product chorismate to isochorismate (Wildermuth *et al.*, 2001).

Systemic acquired resistance (SAR) is an inducible defense response of plants that is triggered by a localized microbial attack in leaves and within a few days renders the whole foliage immune to subsequent pathogen infection (Shah and Zeier, 2013). It is well established that SA is an important resistance-enhancing metabolite in the systemic immunity of dicotyledonous plants such as Arabidopsis or tobacco, because plants that are unable to accumulate SA show strongly attenuated SAR (Gaffney *et al.*, 1993; Nawrath and Métraux, 1999; Bernsdorff *et al.*, 2016). However, previous grafting experiments with tobacco and time-course studies with *Pseudomonas syringae*-inoculated cucumber have suggested that SA is not the primary SAR inducer that transmits resistance signaling from inoculated leaves to distantly located systemic leaves. Instead, the existence of another SAR signal has been proposed (Rasmussen *et al.*, 1991; Vernooij *et al.*, 1994). More recent research has provided evidence that the lysine-derived metabolite *N*-hydroxypipecolic acid (NHP) is a critical activator of systemic immunity in Arabidopsis and that its endogenous biosynthesis is indispensable for pathogen-induced SAR (Hartmann *et al.*, 2018). In addition, a function for NHP as a mobile inducer of SAR has been suggested (Chen *et al.*, 2018).

NHP is not synthesized in unstressed Arabidopsis plants but markedly accumulates systemically in the leaves of pathogen-inoculated plants (Hartmann *et al.*, 2018). Three pathogen-inducible genes are involved in the biosynthesis of NHP in Arabidopsis: *AGD2-LIKE DEFENSE RESPONSE PROTEIN1* (*ALD1*), SAR-DEFICIENT4 (*SARD4*), and *FLAVIN-DEPENDENT-MONOOXYGENASE1* (*FMO1*) (Hartmann and Zeier, 2019). ALD1 functions as an l-Lys-α-aminotransferase and thus deaminates l-Lys to ɛ-amino-α-ketocaproic acid, which spontaneously cyclizes to dehydropipecolic acid intermediates. These are reduced by SARD4 to pipecolic acid (Pip) (Ding *et al.*, 2016; Hartmann *et al.*, 2017). The non-protein amino acid Pip (homoproline) accumulates to high levels in locally inoculated and systemic leaf tissue of Arabidopsis, and this accumulation is necessary for SAR (Návarová *et al.*, 2012). Pip is also synthesized by an ALD1-/SARD4-mediated l-Lys catabolic pathway in the fir clubmoss *Huperzia serrata* (Xu *et al.*, 2018). Moreover, Pip has been shown to accumulate in several angiosperms other than Arabidopsis upon contact with a pathogen—for example, in soybean inoculated with the fungi *Rhizoctonia solani* and *Fusarium virguliforme* (Aliferis *et al.*, 2014; Abeysekara *et al.*, 2016), in tobacco mosaic virus- or *P. syringae*-inoculated tobacco (*Nicotiana tabacum*) (Pálfi and Dézsi, 1968; Vogel-Adghough *et al.*, 2013; Ádám *et al.*, 2018), in rice infected with the blast fungus *Magnaporthe oryzae* (Pálfi and Dézsi, 1968), and in *P. syringae*-infected barley (Lenk *et al.*, 2019).

In Arabidopsis, elevated Pip was found to trigger SAR in an *FMO1*-dependent manner (Návarová *et al.*, 2012; Bernsdorff *et al.*, 2016). The monooxygenase FMO1, which was first identified as an essential player in pathogen-induced SAR in Arabidopsis (Mishina and Zeier, 2006), was more recently shown to catalyze the *N*-hydroxylation of Pip to NHP (Hartmann *et al.*, 2018; Chen *et al.*, 2018). When exogenously supplied to Arabidopsis, NHP potently activated SAR to bacterial (*P. syringae*) and oomycete (*Hyaloperonospora arabidopsidis*) infections and overrode the SAR incompetence of *fmo1* mutant plants (Hartmann *et al.*, 2018). These findings demonstrated that NHP, as the end product of the described three-step l-Lys catabolic pathway, functions as the active, SAR-inducing metabolite of this immune pathway. When locally applied to lower leaves, NHP also induced resistance in upper leaves, suggesting a function in leaf-to-leaf signal transmission in SAR (Chen *et al.*, 2018).

The natural occurrence of NHP in living organisms was described for the first time in Arabidopsis (Hartmann *et al.*, 2018). Since the NHP biosynthetic precursor Pip is naturally present in many different angiosperms (Broquist, 1991; Zeier, 2013), and genes homologous to Arabidopsis *FMO1* exist in monocots and dicots (Hartmann *et al.*, 2018; Chen *et al.*, 2018), it is conceivable that NHP biosynthesis and its function in SAR are conserved across angiosperms. In Arabidopsis, the induction of an effective SAR response by NHP requires its interplay with SA (Bernsdorff *et al.*, 2016; Hartmann *et al.*, 2018; Hartmann and Zeier, 2019). However, it is unclear so far whether the molecular mechanisms of SAR elucidated for Arabidopsis and other dicots, as well as the function of SA in systemic immunity, hold true for monocot species (Balmer *et al.*, 2013; Dey *et al.*, 2014).

In this study, we show that the pathogen-inducible biosynthesis of NHP and its ability to induce plant SAR is present in distinct monocotyledonous and dicotyledonous plant species. Our results generalize the existence of an NHP-associated immune pathway in plants and highlight mechanistic similarities in the SAR of dicot and monocot plants.

## Materials and methods

### Plant material and growth conditions

*Arabidopsis thaliana* accession Col-0 plants were grown as outlined previously (Hartmann *et al.*, 2018). Plants of six species (*Cucumis sativus* cv. Wisconsin SMR58, *Nicotiana tabacum* cv. Xanthi, *Solanum lycopersicum* cv. Heinz1706, *Glycine max* cv. Maple Arrow, *Brachypodium distachyon* accession BD21, and *Hordeum vulgare* cv. Ingrid) were grown in environmentally controlled growth chambers on a mixture of soil, vermiculite, and sand with a 16 h/8 h light/dark cycle (see Supplementary Table S1 at *JXB* online for specific growth conditions). Plants of *S. lycopersicum* cv. M82 infected with *Phytophthora infestans* were cultivated as described previously (de Vries *et al.*, 2017).

### Cultivation of pathogens

*Pseudomonas syringae* pv. *lachrymans* strain 814/98 (*Psl*) (Słomnicka *et al.*, 2018*), P. syringae* pv*. tomato* (*Pst*) DC3000, *P. syringae* pv. *maculicola* ES4326 (*Psm*), *Psm* carrying the *Photorhabdus luminescens* luxCDABE operon (*Psm lux*) (Hartmann *et al.*, 2018), and *Pseudomonas savastanoi* pv. *glycinea* (DSM 50267) (*Psg*) were grown at 28 °C in King’s B medium containing the appropriate antibiotics (*Pst* DC3000 and *Psm*, 50 μg l^–1^ rifampicin; *Psm lux*, 50 μg l^–1^ of rifampicin and 25 μg l^–1^ kanamycin; *Psl* and *Psg*, no antibiotics). *Pseudomonas syringae* pv. *tabaci* (DSM 1856) (*Pstb*) was grown at 18 °C in King’s B medium without antibiotics (Vogel-Adghough *et al.*, 2013). *Xanthomonas translucens* (DSM 18974) was grown at 28 °C on peptone-sucrose agar (20 g l^–1^ sucrose, 5 g l^–1^ peptone, 0.5 g l^–1^ K_2_HPO_4_, 0.25 g l^–1^ MgSO_4_x7H_2_O, 15 g l^–1^ agar). *Phytophthora infestans* strain IPO-C was cultured in the dark at 18 °C on rye sucrose agar as described previously (de Vries *et al.*, 2017). *Magnaporthe oryzae* strain Guy11 was cultivated on cornmeal agar supplemented with a nitrate salt solution at 18 °C (9 h light) as reported by Parker *et al.* (2008). The *M. oryzae* isolate TH6772 was cultured on oatmeal agar plates (20 g l^–1^ agar, 2 g l^–1^ yeast extract, 10 g l^–1^ starch, 30 g l^–1^ oat flakes) at 23 °C in the dark (Delventhal *et al.*, 2017).

### Plant inoculation experiments

For baterial inoculation experiments, log-phase cultures of the respective bacteria were grown overnight under constant shaking (240 rpm). The cultures were washed three times with 10 mM MgCl_2_ and diluted to different final optical density levels at 600 nm (OD_600_) for leaf inoculations. Mock-control plants were inoculated likewise with 10 mM MgCl_2_ solution, unless stated otherwise.

#### Arabidopsis thaliana

Leaves of 5-week-old Arabidopsis Col-0 plants were infiltrated with a suspension of *Psm lux* (OD_600_=0.001) as described previously (Hartmann *et al.*, 2018).

#### Cucumis sativus

Three-week-old *C. sativus* plants were spray-inoculated with a suspension of *Psl* of OD_600_=0.2 (supplemented with 0.005% of the surfactant Silwet® L-77) applied to the abaxial side of the first true leaf. For the collection of *C. sativus* phloem sap, locally inoculated (first true) or distal (second true) leaves were cut and the escaping sap was collected at the site of the petiole by using filter paper. Filter paper soaked with ~10 µl of phloem sap was transferred into 100 μl MeOH/H_2_O (80:20, v/v) for metabolite extraction. Leaf discs (0.5 cm^2^) and phloem sap samples for subsequent metabolite analyses were collected at 1, 3, and 5 days post-inoculation (dpi), frozen in liquid nitrogen, and stored at –80 °C (*n*=5). For the resistance assays, discs of inoculated *C. sativus* leaves were harvested at 3 dpi (*n*=4) and 6 dpi (*n*=4).

#### Nicotiana tabacum

*Nicotiana tabacum* plants 4 to 5 weeks old were carefully pressure-infiltrated from the abaxial side of the leaves with a *Pstb* or *Psm* suspension, using a needleless syringe. For metabolite analyses, discs (1.0 cm^2^) of *N. tabacum* leaves inoculated with *Pstb* (OD_600_=0.005) were harvested from 1 to 3 dpi, frozen in liquid nitrogen, and stored at –80 °C (*n*=3). For resistance assays, leaf discs of *Pstb* [OD_600_=0.001 (*n*=12)] and *Psm* [OD_600_=0.001 (*n*≥5)] inoculated plants were sampled at 3 dpi.

#### Glycine max

Three-week-old *G. max* plants at growth stage 12–13 (BBCH scale) were pressure-infiltrated as described above with a *Psg* suspension (OD_600_=0.1). Samples of inoculated *G. max* leaves were harvested at 9 dpi as described above.

#### Solanum lycopersicum

For bacterial inoculations, 4-week-old *S. lycopersicum* cv. Heinz1706 plants were pressure-infiltrated with a suspension of *Pst* DC3000 (OD_600_=0.005) from the abaxial side of fully expanded leaves, using a needleless syringe. Sampling for metabolite analyses was performed at 1 dpi and 2 dpi (*n*=3) as described above. For oomycete inoculations of *S. lycopersicum* cv. M82, a suspension of *P. infestans* containing 50 spores µl^–1^ was used for droplet inoculation (10 µl droplet applied to each leaf) of 4-week-old plants grown in closed vessels (de Vries *et al.*, 2017). Mock controls were treated likewise with sterile H_2_O. For the metabolite analyses of *P. infestans-* and mock-inoculated *S. lycopersicum* cv. M82 leaves, samples were harvested at 1 dpi and 2 dpi (*n*≥4) as described above.

#### Brachypodium distachyon

For metabolite analyses, 5-week-old *B. distachyon* plants were inoculated with a suspension of *X. translucens* (OD_600_=0.5). Sampling (one plant per sample) was carried out at 1, 3, and 5 dpi (*n*=3). Leaf samples were frozen in liquid nitrogen and stored at –80 °C. For resistance assays, 3-week-old *B. distachyon* plants were inoculated with a bacterial suspension of OD_600_=0.005. The assessment of resistance was performed at 3 dpi (*n*=9). In both experimental setups, the bacterial suspension was infiltrated from the adaxial site of the leaves, using a needleless syringe.

For the inoculation of *B. distachyon* with *M. oryzae*, a suspension of conidia (50 spores μl^–1^) was applied to 3- to 4-week-old plants by airbrush spray-inoculation. To prepare the spore suspension, spores were isolated within 15 days of growth on plates by washing the mycelium with sterile H_2_O and scraping off the spores using a coverslip. The resulting solution was filtered through Miracloth (Merck Millipore, Billerica, MA, USA) and centrifuged for 4 min at 2200 *g*. After resuspending the pellet in 3 ml sterile H_2_O, the concentration of spores was determined using a Neubauer chamber (NanoEnTek. Inc., Seoul, Korea). Plants were sprayed until they were covered evenly with fine droplets. After inoculation, the plants were maintained hermetically sealed in autoclave bags as described by Parker *et al.* (2008). For metabolite analyses, one plant per sample was harvested at 2, 3, and 4 dpi (*n*≥4). The assessment of resistance against *M. oryzae* was performed at 4 dpi.

#### Hordeum vulgare

*Magnaporthe oryzae* conidia were harvested from oatmeal agar plates after incubation for 2 weeks under UV-A light and the suspension was adjusted to 250 spores μl^–1^. Primary leaves of barley plants were spray-inoculated with a spore suspension containing 1 g l^−1^ gelatin and 0.5 ml l^−1^ Tween. After spray-inoculation, plants were kept for 24 h at 24 °C and 100% relative humidity in the dark and then transferred to growth chamber conditions. Mock-control plants were spray-inoculated with H_2_O containing 1 g l^−1^ gelatin and 0.5 ml l^−1^ Tween. Inoculated *H. vulgare* leaves were sampled at 5 dpi (*n*=3).

### Plant treatments with *N*-hydroxypipecolic acid

#### Arabidopsis thaliana

NHP treatment of Arabidopsis via the root was performed by pipetting 10 ml of aqueous NHP solutions of different concentrations on to the soil of individually cultivated 5-week-old plants (Hartmann *et al.*, 2018). For spray treatments of Arabidopsis leaves, the whole rosette of 5-week-old plants was sprayed with a 1 mM aqueous solution of NHP, supplemented with 0.005% Silwet® L-77, until small droplets formed evenly on the leaf surfaces (~0.5 ml solution per plant). For treatments by leaf infiltration, three rosette leaves per plant were infiltrated with 1 mM NHP solution. Application of H_2_O by the respective method served as a control treatment for all treatment types. Leaves were inoculated with *Psm lux* 1 day after the NHP treatments.

#### Nicotiana tabacum

Treatments of tobacco plants with NHP were performed following the procedure described by Vogel-Adghough *et al.* (2013) for Pip solutions. Individually cultivated plants were watered with 10 ml of a 1 mM aqueous solution of NHP by pipetting. As a control treatment, 10 ml H_2_O was applied in the same manner. Inoculation of leaves with *Pstb* or *Psm* was performed 24 h after the application of the NHP treatment.

#### Cucumis sativus

Approximately 1 ml of 1 mM NHP solution was directly injected into the plant stem using a syringe with a needle. An injection of sterile H_2_O served as a control. One day later, the leaves were spray-inoculated with a *Psl* suspension.

#### Brachypodium distachyon

1 mM NHP solution was syringe-infiltrated thoroughly from the adaxial site into the leaves of *B. distachyon* until the entire leaf area appeared to be soaked. Plants were inoculated with *X. translucens* or *M. oryzae* 1 day later. Infiltration with H_2_O was used as a control treatment.

### Assessment of resistance

The resistance of Arabidopsis against *Psm lux* infection was determined as described previously (Hartmann *et al.*, 2018). For other bacterial growth assays, leaf discs were cut out of inoculated leaf material and homogenized in 10 mM MgCl_2_. Each sample was diluted in 10 mM MgCl_2_ (1:40 000 for *Psl*/*C. sativus*; 1:20 000 for *Pstb*/*N. tabacum*; 1:5000 for *Psm*/*N. tabacum*; 1:400 000 for *X. translucens*/*B. distachyon*), plated on agar plates (1.5% agar) containing the above-described media, and incubated for 2–4 days at 28 °C before the number of bacterial colonies was determined.

To assay the resistance of *B. distachyon* to *M. oryzae,* the youngest fully grown leaves at the time of inoculation were removed and bleached using an ethanol–chloroform solution (4:1) containing 1.5% trichloroacetic acid. After a minimum of 2 days, the bleaching solution was removed with H_2_O and the leaves were photographed on a light table. The proportion of the necrotic leaf area was measured using ImageJ (https://imagej.nih.gov/ij/).

### Analysis of plant metabolites by gas chromatography-mass spectrometry

To determine the levels of Pip, NHP, SA, SAG, NHP-hexose, and lysine in the leaves of the different species, the gas chromatography-mass spectrometry-based analytical procedure previously established for Arabidopsis was used (detailed in Hartmann *et al.*, 2018; Stahl *et al.*, 2019). For metabolic analyses of cucumber phloem sap, the above-described filter paper extracts were supplemented with 900 µl of MeOH/H_2_O (80:20, v/v) and analysed.

### Analysis of gene expression

The expression of the *C. sativus ALD1* (XM_011651615.1), *FMO1* (XM_004144232.1), and *Actin* (XM_004147305.2) genes was assessed by semi-quantitative RT–PCR analysis. RNA extraction and cDNA synthesis were performed as described by Wang *et al.* (2018). Primers and PCR conditions are listed in Supplementary Table S2. PCRs were performed using GoTaq Green Master Mix (Promega) following the manufacturer’s instructions.

### Statistical analyses and reproducibility

The presented results are derived from experimental datasets consisting of the number of biological replicates indicated in the figure legends and/or in the above sections. For the statistical analyses of bacterial growth data, log_10_-transformed colony-forming units or relative light units values were used. ANOVA with type II sum of squares was applied for multiple comparisons (Bernsdorff *et al.*, 2016). For pairwise comparisons of two distinct treatments, two-tailed Student’s *t*-tests were performed. The presented results were generally confirmed in at least two other independent experiments, except for the barley infection experiment (Fig. 5C), which was conducted once.

## Results

We previously reported the identification of the hitherto unknown plant natural product NHP, its FMO1-mediated biosynthesis from Pip, its pathogen-inducible accumulation, and its biological function as a SAR inducer in the model plant *A. thaliana* (Hartmann *et al.*, 2018). On this basis, we were interested to investigate whether the inducible biosynthesis of NHP and its function in acquired resistance were a common feature in angiosperms. To generalize the occurrence and function of the NHP pathway in plant immunity, we implemented different naturally relevant plant–pathogen interaction systems in the laboratory. We further conducted defense-related metabolite analyses and resistance assays to test whether exogenously applied NHP would induce acquired resistance in species other than Arabidopsis.

### NHP induces SAR in Arabidopsis at low doses and by different treatment modes

**Fig. 1.**
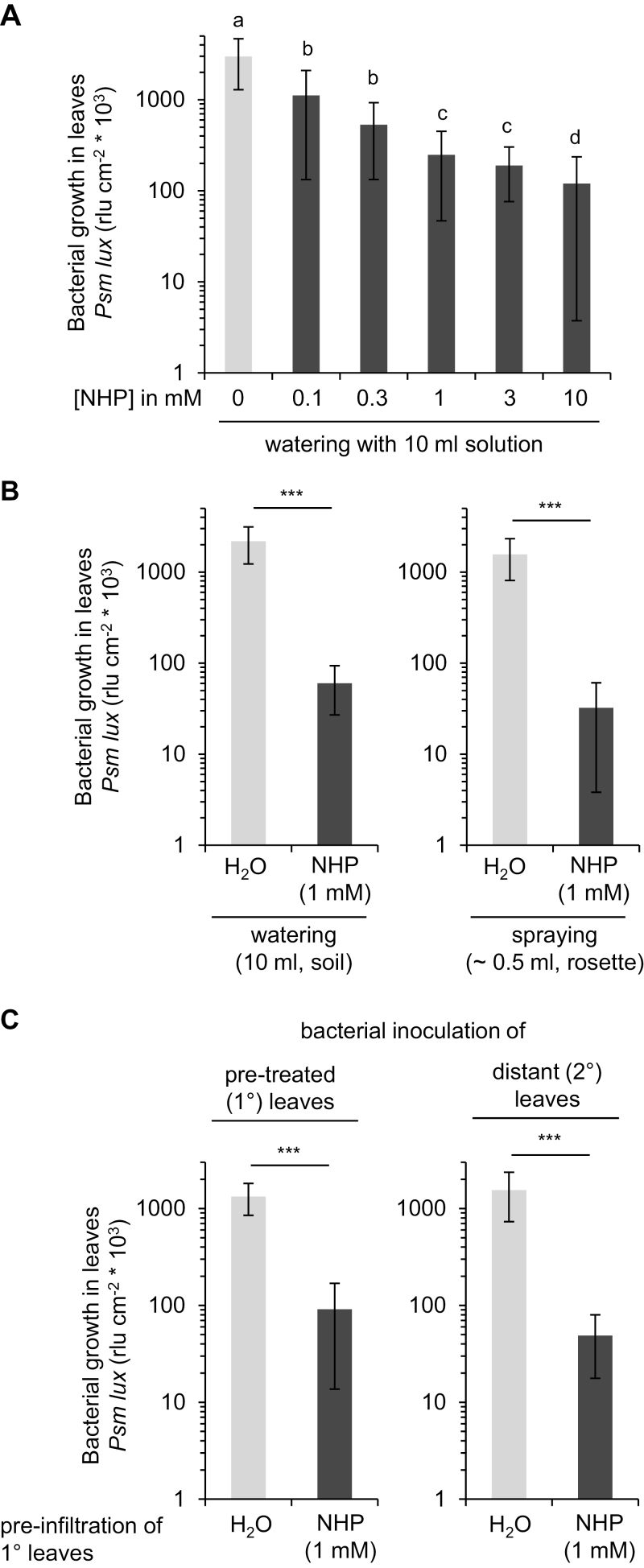
Exogenous NHP induces SAR in Arabidopsis by different treatment modes. (A) Dose dependency of SAR in leaves triggered by treatment of roots with NHP. The cultivation soil of individually grown 5-week-old Arabidopsis Col-0 plants was watered with 10 ml of an aqueous NHP solution at the indicated concentration. Treatment with H_2_O served as the control. One day later, three leaves of each plant were inoculated with the compatible bioluminescent *Pseudomonas syringae* pv. *maculicola* (*Psm*) *lux* strain (OD_600_=0.001). Bacterial numbers in leaves were assessed at 2.5 dpi by measurement of luminescence and are expressed as relative light units (rlu) per cm^2^ leaf area (Hartmann *et al.*, 2018). Data represent the mean ±SD of at least 21 leaf replicates from 7 or more plants. Different letters denote significant differences (*P*<0.05; ANOVA and post-hoc Tukey’s honestly significant difference test). (B) Comparison of soil application and foliar spray treatment of Col-0 plants with NHP solutions. Left panel: Col-0 plants were watered with 10 ml of a 1 mM NHP solution or H_2_O. Right panel: Whole leaf rosettes were sprayed with ~0.5 ml of a 1 mM NHP solution or H_2_O (both supplemented with 0.005% Silwet® L-77) until the adaxial side of the leaves was evenly covered in fine droplets of the solution. One day later, the leaves were inoculated with *Psm lux* and bacterial numbers were quantified at 2.5 dpi. Statistically significant differences between NHP-pretreated and control plants are indicated with asterisks: ***P<0.001 (two-tailed *t* test). (C) Infiltration of individual Col-0 rosette leaves with NHP solution induces SAR in the infiltrated leaves and in distant leaves. Three leaves of a given Col-0 plant were syringe-infiltrated with 1 mM NHP solution or H_2_O. One day later, either the pretreated (1°) leaves or three distant (2°) leaves were inoculated with *Psm lux* and bacterial numbers were quantified at 2.5 dpi (***P<0.001; two-tailed *t* test).

In our previous study on the role of NHP in SAR, we observed that exogenous NHP applied via the soil to individual Arabidopsis plants in doses of 10 µmol (i.e. 10 ml of a 1 mM NHP solution) induced a strong SAR effect in the foliage (Hartmann *et al.*, 2018). To get more information about the dose dependency of this NHP-triggered resistance response, we supplied NHP solutions at concentrations ranging from 0.1 to 10 mM to individual Arabidopsis plants by the same method. We then inoculated leaves of the plants with the compatible bacterial strain *Psm lux* 1 day later and scored bacterial numbers at 2.5 dpi. Watering with a concentration of 0.1 mM NHP attenuated bacterial growth by ~3-fold, indicating that doses of NHP as low as 1 µmol applied via the roots induce a detectable SAR effect in Arabidopsis (Fig. 1A). Increases in the concentration of NHP up to 1 mM gradually enhanced this SAR response to more than one log difference compared with the control treatment, and increases to 10 mM resulted in further modest improvements of the SAR effect (Fig. 1A). Watering the soil with 1 mM NHP solution generally resulted in a stable SAR effect that reduced bacterial growth in the leaves by 10–50-fold (Fig. 1A, B; Hartmann *et al.*, 2018). 

Next, to test whether treating leaves directly with NHP would yield similar resistance effects, we supplied NHP to the foliage by spraying and by infiltration. Spraying the whole leaf rosette with 1 mM NHP solution induced strong resistance and attenuated bacterial growth in infected leaves by a factor of ~50-fold compared with control plants (Fig. 1B). Because the overall volume applied to a single plant was ~0.5 ml for the spray application, the effective dose of NHP to induce a strong SAR was ~0.5 µmol per plant for leaf treatments. Moreover, when individual leaves were pre-infiltrated with 1 mM NHP solution, the same leaves acquired resistance to subsequent *Psm lux* infection, and bacterial growth at 2.5 dpi was again reduced by more than one order of magnitude (Fig. 1C). Notably, the untreated distant leaves of plants that had been pre-infiltrated with NHP in other leaves showed induction of a very strong SAR (Fig. 1C), corroborating the previously established critical role of NHP in SAR long-distance communication (Chen *et al.*, 2018; Hartmann *et al.*, 2018). Together, these results show that NHP applied by different modes of treatment is able to induce SAR and exerts a strong biological activity in doses lower than 1 µmol.

### NHP accumulates systemically in the foliage and in phloem sap of cucumber, and induces SAR in this species

Arabidopsis has developed into the primary model plant for SAR research in the past two to three decades (e.g. Fu and Dong, 2013; Shah and Zeier, 2013). In the most studied Arabidopsis accession, Col-0, the basal levels of the SAR-associated metabolites SA, Pip, and NHP are generally low (SA, Pip) or undetectable (NHP) in unstressed plants, and rise strongly during the course of infection with *P. syringae* in both locally inoculated and distal systemic leaf tissue (Hartmann and Zeier, 2019). In addition, cucumber was used in a series of studies as a model to investigate the molecular mechanisms and physiology of the SAR response (e.g. Métraux *et al.*, 1990; Rasmussen *et al.*, 1991). Cucumber plants inoculated with compatible lesion-inducing bacterial, fungal, or viral phytopathogens in one leaf show induction of robust SAR and systemic defense responses in distal leaves. Cucumber and other species of Cucurbitaceae also release phloem sap from cut petiole or stem tissues that can readily be analyzed for its molecular content, including potentially phloem-mobile SAR-active metabolites (Métraux *et al.*, 1990; Smith-Becker *et al.*, 1998).

**Fig. 2.**
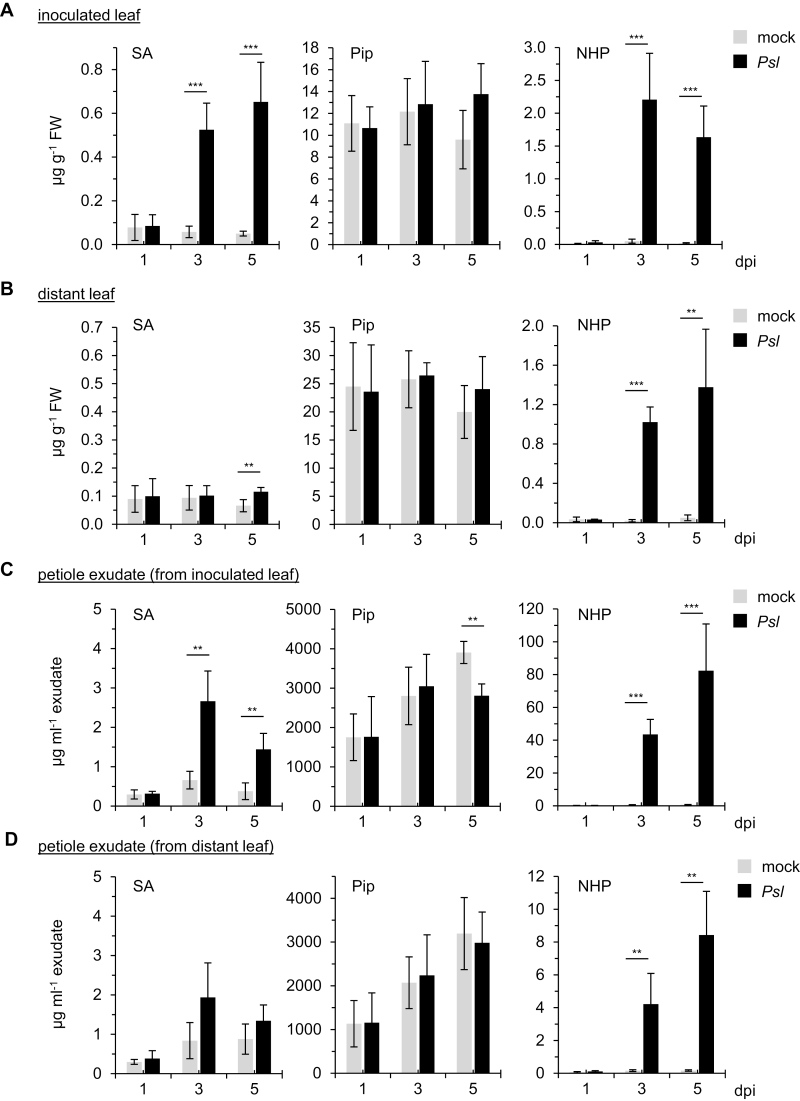
NHP systemically accumulates in the leaves and petiole sap of *Cucumis sativus* upon inoculation with *Pseudomonas syringae* pv. *lachrymans*. (A) Levels of SA, Pip, and NHP in *Cucumis sativus* cv. Wisconsin SMR58 leaves inoculated with *P. syringae* pv. *lachrymans* (*Psl*) and in mock-treated control leaves. The first grown true leaves of plants at the two-leaf stage were spray-inoculated with a suspension of *Psl* (OD_600_=0.2, containing 0.005% Silwet® L-77) or with 0.005% Silwet® L-77 dissolved in 10 mM MgCl_2_ (mock treatment). Samples of treated leaves were collected at 1, 3, or 5 dpi. Metabolite levels are given in µg g^–1^ leaf FW and represent the mean ±SD of four or five biological replicates (*n*=4 or 5). (B) Levels of SA, Pip, and NHP in the second, distal leaves of cucumber plants inoculated on the first leaves with *Psl* or infiltrated with 10 mM MgCl_2_ as mock treatment (*n*=4 or 5). (C) Concentrations of SA, Pip, and NHP in petiole sap collected from *Psl*-inoculated or mock-treated *C. sativus* leaves. Metabolite concentrations are given in µg ml^–1^ collected petiole exudate (*n*=4 or 5). (D) Concentrations of SA, Pip, and NHP in petiole sap collected from the second, distal leaves of cucumber plants in which the lower, first leaves had been *Psl*-inoculated or mock-treated (*n*=4 or 5). Statistically significant differences between samples from pathogen-inoculated and mock-treated leaves at a given time point are indicated with asterisks: ***P<0.001, **P<0.01, *P<0.05 (two-tailed *t*-test).

We inoculated *C. sativus* cv. Wisconsin SMR58 plants at the two-leaf stage in their first true leaf with the causal agent of angular leaf spot, *Psl* (Słomnicka *et al.*, 2018), a pathogen that elicits systemic defense responses in cucumber (Mölders *et al.*, 1994). SA and its β-glucoside SAG accumulated significantly in the *Psl*-inoculated first leaves at 3 dpi and 5 dpi, whereas the systemic accumulation of SA in the distal leaves was modest, and that of SAG was not significant (Fig. 2A, B; Supplementary Fig. S1A). The basal levels of Pip were between 10 and 25 µg g^–1^ fresh weight (FW) in uninfected cucumber leaves (Fig. 2A, B), markedly higher than the basal levels of Pip in leaves of Arabidopsis Col-0 (~0.5 µg g^–1^ FW; Návarová *et al.*, 2012). In contrast to the response in Arabidopsis, pathogen inoculation did not result in increases of Pip either locally or systemically (Fig. 2A, B). Consistent with this constitutively strong accumulation of Pip, transcripts of the *C. sativus ALD1* gene were readily detectable in the leaves of control plants (Supplementary Fig. S2). However, in contrast to the amount of Pip in uninfected cucumber plants, the levels of NHP were low (<0.05 µg g^–1^ FW). Notably, NHP accumulated markedly in both the inoculated (2.2 µg g^–1^ FW) and distant (1.4 µg g^–1^ FW) leaves in response to *Psl* at 3 dpi and 5 dpi (Fig. 2A, B). The pathogen-induced accumulation of NHP was paralleled by a further increase of *ALD1* transcripts in *Psl*-inoculated leaves at 3 dpi (Supplementary Fig. S2). Moreover, transcripts of the *C. sativus FMO1* gene, which were not detected in the leaves of control plants, accumulated at 3 dpi and 5 dpi (Supplementary Fig. S2). As previously reported for Arabidopsis (Hartmann and Zeier, 2018), we also detected a putative NHP-hexose conjugate in extracts from *Psl*-inoculated cucumber leaves (Supplementary Fig. S1B). Together, these results show that cucumber possesses an intact and pathogen-inducible NHP biosynthetic pathway.

SAR long-distance signal propagation from one leaf to another is supposedly mediated by signal-active, phloem-mobile metabolites (Shah and Zeier, 2013). We collected phloem sap exuded from the petioles of cut local (first true) or distant (second true) leaves following *Psl* inoculation or mock treatment and analyzed the contents of SAR-relevant metabolites (Fig. 2C, D). Both SA (~2 µg ml^–1^) and SAG (~8 µg ml^–1^) were significantly enriched in the phloem sap of *Psl-*inoculated local leaves, and a pathogen-induced increase of SAG content was also detected in phloem sap collected from distant leaves (Fig. 2C, D; Supplementary Fig. S1A). Notably, the constitutive levels of Pip in phloem sap were in the range 1–4 mg ml^–1^ (~8–30 mM) and therefore about three orders of magnitude higher than the contents of SA/SAG in the petiole sap from inoculated leaves. As was observed for the leaf contents, *Psl* inoculation did not result in further increases in these prominent levels of Pip in phloem sap from local or distant leaves (Fig. 2C, D). By contrast, the levels of NHP were barely detectable in phloem sap from mock-treated control plants. Notably, NHP levels increased largely in the sap of both local (~80 µg ml^–1^) and distant (~8 µg ml^–1^) leaves upon *Psl* inoculation (Fig. 2C, D). Together, these results show that localized inoculation of a pathogen triggers a strong local and systemic accumulation of NHP in both the leaves and the leaf phloem sap of cucumber. This accumulation is greater than the increases of SA and SAG. This observation supports a function of NHP in SAR-associated phloem-based long-distance signaling. The NHP biosynthetic precursor Pip is constitutively present in high amounts in both leaves and phloem sap of cucumber.

**Fig. 3.**
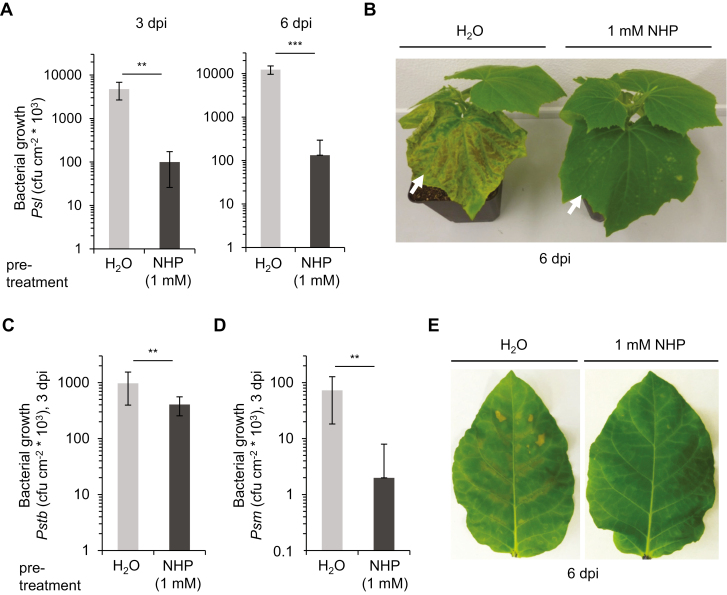
*Cucumis sativus* and *Nicotiana tabacum* plants pretreated with a micromolar dose of NHP acquire resistance to bacterial infection. (A) Growth of *Pseudomonas syringae* pv. *lachrymans* (*Psl*) in *C. sativus* leaves. Three-week-old cucumber plants were pretreated with NHP by injecting 1 ml of 1 mM aqueous NHP solution into the stem. Injection of H_2_O served as a control. One day later, leaves were spray-inoculated with *Psl* (OD_600_=0.2). Bacterial numbers in leaves, expressed as colony-forming units (cfu) per cm^2^ leaf surface, were scored at 3 dpi and 6 dpi. Data represent the mean ±SD of five biological replicates (*n*=5). Statistically significant differences between bacterial growth in the leaves of NHP- and H_2_O-pretreated plants are indicated with asterisks: ***P<0.001, **P<0.01 (two-tailed *t*-test). (B) Representative disease symptoms shown by *C. sativus* plants pretreated with H_2_O or 1 mM NHP at 6 days post spray-inoculation with *Psl.* Arrows indicate the inoculated first leaves. (C) Growth of *P. syringae* pv. *tabaci* (*Pstb*) in *N. tabacum* cv. Xanthi leaves. Plants were pretreated with NHP by the addition of 10 ml of 1 mM NHP to the cultivation soil. Control plants were watered with 10 ml H_2_O. Leaves were infiltrated with a suspension of *Pstb* (OD_600nm_=0.001) 1 day later; bacterial growth was assessed at 3 dpi. Data represent the mean ±SD of at least 11 biological replicates (*n*=11). (D) Growth of *P. syringae* pv. *maculicola* (*Psm*) in leaves of *N. tabacum* cv. Xanthi after infiltration with a suspension of OD_600_=0.001. The experimental setup was as described in C (*n*=11). (E) Representative disease symptoms shown by *Pstb*-inoculated *N. tabacum* leaves from plants pretreated with 10 ml H_2_O or 1 mM NHP at 6 dpi.

To test whether elevated levels of NHP trigger acquired resistance to *Psl* infection in cucumber, we injected an aqueous solution of NHP (1 mM) into the epicotylar *C. sativus* stem as an inducing pretreatment and challenge-inoculated the first true leaves with *Psl* 1 day later. Plants pretreated with NHP showed strongly enhanced resistance to *Psl* leaf infection compared with H_2_O-pretreated control plants (Fig. 3A, B). NHP-pretreated plants hosted 50- and 100-fold lower amounts of bacteria in the *Psl*-inoculated leaves at 3 dpi and 6 dpi, respectively (Fig. 3A). In addition, the yellowish and chlorotic disease symptoms observed on leaves of control cucumber plants were greatly diminished as a consequence of NHP pretreatment (Fig. 3B). Together, these results indicate that NHP is able to induce SAR in cucumber.

### Biosynthesis and resistance function of NHP in other dicotyledonous plant species

**Fig. 4.**
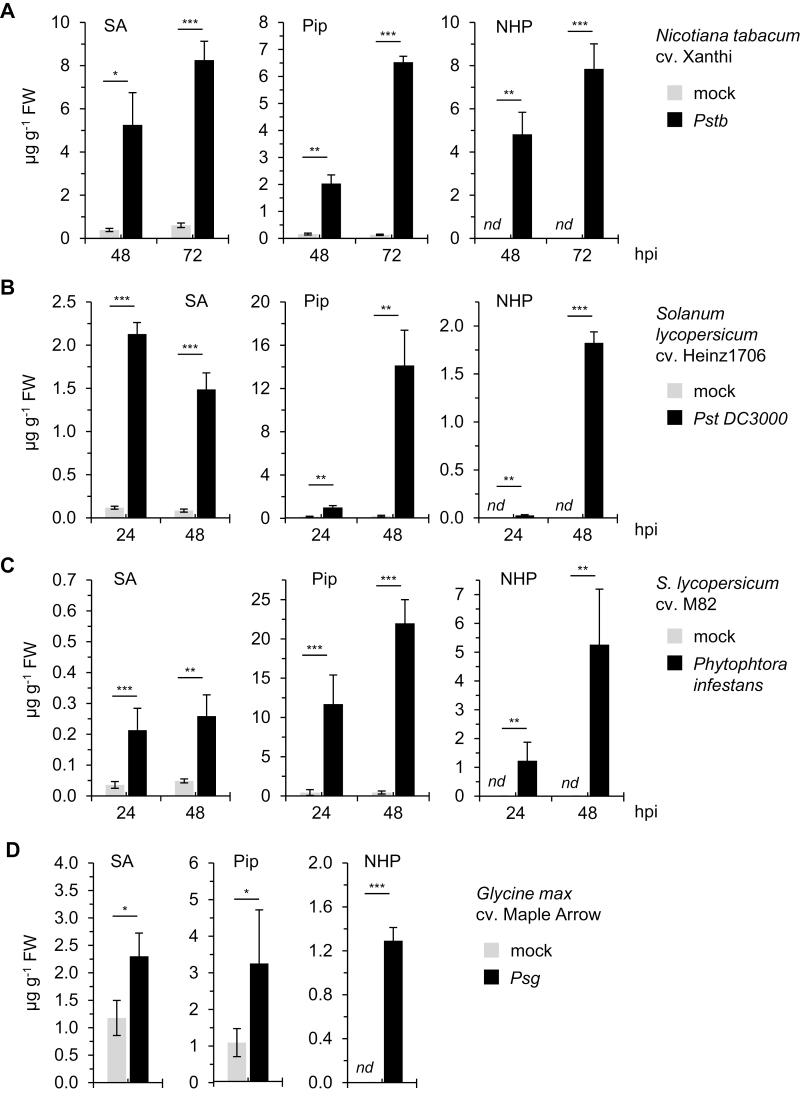
NHP accumulates in leaves of *Nicotiana tabacum*, *Solanum lycopersicum*, and *Glycine max* inoculated with bacterial and/or oomycete pathogens. (A) Levels of SA, Pip, and NHP in leaves of *N. tabacum* cv. Xanthi inoculated with *Pseudomonas syringae* pv. *tabaci* (*Pstb*) and in leaves of mock-treated control plants. Leaves of 4-week-old plants were infiltrated with a bacterial suspension at OD=0.005 and samples were collected at 48 and 72 h post inoculation (hpi). Leaf metabolite levels are given in µg g^–1^ FW (*n*=3). *nd*, Not detected. Other details are as described in Fig. 2A. (B) Levels of SA, Pip, and NHP in leaves of *S. lycopersicum* cv. Heinz1706 inoculated with *P. syringae* pv. *tomato* (*Pst*) DC3000 and in leaves of mock-treated control plants. Leaves of 3-week-old plants were infiltrated with a bacterial suspension of OD=0.005 and samples were collected at 24 and 48 hpi (*n*=3). (C) Levels of SA, Pip, and NHP in leaves of *S. lycopersicum* cv. M82 inoculated with *P. infestans* IPO-C and in leaves of mock-treated control plants. Leaves of 3-week-old plants were inoculated with droplets containing 50 spores µl^–1^. Samples were collected at 24 and 48 hpi (*n*≥4). (D) Levels of SA, Pip, and NHP in leaves of *G. max* cv. Maple Arrow inoculated with *Pseudomonas savastanoi* pv. *glycinea* (*Psg*) and in leaves of mock-treated control plants. Leaves of 3-week-old plants were infiltrated with a bacterial suspension of OD=0.1 and samples were collected at 9 dpi (*n*=4). Data are presented as mean ±SD. Statistically significant differences between samples from pathogen-inoculated and mock-treated leaves at a given time point are indicated with asterisks: ***P<0.001, **P<0.01, *P<0.05 (two-tailed *t*-test).

Pip, the direct biosynthetic precursor of NHP, occurs as a natural product in a wide array of angiosperms (Broquist, 1991; Hartmann and Zeier, 2018). Several studies have demonstrated that Pip also accumulates in the foliage of various dicotyledonous species other than Arabidopsis in response to pathogen inoculation. For example, biosynthesis of Pip is induced in *N. tabacum* leaves in response to infection by the causal agent of tobacco wildfire disease, *Pstb*, and by tobacco mosaic virus (Pálfi and Dézsi, 1968; Vogel-Adghough *et al.*, 2013; Ádám *et al.*, 2018). We examined whether NHP is detectable and accumulates, alongside Pip and SA, in *Pstb*-inoculated tobacco leaves. The basal levels of SA and Pip were low in the leaves of control (mock-treated) *N. tabacum* cv. Xanthi plants, but their biosynthesis was significantly increased in response to *Pstb* inoculation. In addition, NHP, which was not detected in the leaves of mock-treated tobacco plants, accumulated substantially in leaves of *Pstb-*inoculated plants, to amounts of ~5 and 8 µg g^–1^ FW at 48 and 72 h post inoculation (hpi), respectively (Fig. 4A). NHP also accumulated in *S. lycopersicum* cv. Heinz1706 leaves from non-detectable basal levels to 1.8 µg g^–1^ FW at 48 h after inoculation with the bacterial speck pathogen *Pst* DC3000 (Fig. 4B). Similarly, inoculation of *S. lycopersicum* cv. M82 leaves with the oomycete *P. infestans*, the causal agent of tomato late blight, resulted in the accumulation of NHP to ~5 µg g^–1^ FW at 48 hpi (Fig. 4C). In both interactions, tomato leaves started to synthesize NHP from ~24 hpi onwards and showed markedly induced accumulation of Pip and SA alongside the biosynthesis of NHP (Fig. 4B, C).

Previous studies also indicated the inducible accumulation of Pip in soybean plants inoculated with the fungal pathogens *Rhizoctonia solani* and *Fusarium virguliforme* (Aliferis *et al.*, 2014; Abeysekara *et al.*, 2016). Consequently, we determined metabolite levels in *G. max* cv. Maple Arrow leaves inoculated with the bacterial blight pathogen *Psg*. Leaves of uninoculated soybean plants contained constitutive levels of both SA and Pip in the range of 1 µg g^–1^ FW, whereas NHP was not detectable. *Psg* inoculation resulted in a significant elevation of the levels of SA and Pip, as well as a noticeable accumulation of NHP to ~1.3 µg g^–1^ FW (Fig. 4D).

Previous findings indicate that feeding of Pip to *N. tabacum* plants enhances resistance to leaf infection by *Pstb* and tobacco mosaic virus (Vogel-Adghough *et al.*, 2013; Ádám *et al.*, 2018). We therefore tested whether exogenous NHP would induce acquired resistance of tobacco to bacterial infection by employing the soil-watering method of NHP pretreatment (Hartmann *et al.*, 2018), which was used previously for supplementing *N. tabacum* plants with Pip (Vogel-Adghough *et al.*, 2013). NHP-pretreated plants showed a significant restriction of *Pstb* growth after challenge inoculation and also showed fewer disease symptoms compared with naive plants, indicating that NHP significantly induces resistance to this bacterial strain (Fig. 3C, E). In experiments in which the same doses of Pip and NHP were supplied to different plants, NHP pretreatment reduced disease severity even more markedly than Pip feeding (Supplementary Fig. S3). We also inoculated *N. tabacum* cv. Xanthi plants with *Psm*, which infects Arabidopsis and causes yellowish and chlorotic symptoms on leaves (Mishina and Zeier, 2007). The *Psm* strain was able to grow in tobacco leaf tissue, but its multiplication in naive plants was approximately one order of magnitude lower than that of the compatible *Pstb* strain at 3 dpi, and the interaction remained essentially symptomless (Fig. 3C, D). Nevertheless, NHP pretreatment strongly attenuated the growth of *Psm* in *N. tabacum* cv. Xanthi leaves (Fig. 3D). Together, these data show that NHP effectively induces resistance of tobacco to bacterial pathogen infection.

In sum, our results show that NHP biosynthesis is undetectable in unstressed plants of the dicotyledonous species we have investigated in this and previous studies. However, NHP significantly accumulates in leaves of members of the Brassicaceae (Arabidopsis; Hartmann *et al.*, 2018), Cucurbitaceae (cucumber), Solanaceae (tobacco, tomato), and Fabaceae (soybean) in response to bacterial and/or oomycete infection (Figs 2, 4). In addition, our findings indicate that low micromolar doses of NHP activate acquired resistance in Arabidopsis (Fig. 1; Hartmann *et al.*, 2018), cucumber, and tobacco (Fig. 3) to bacterial or oomycete infection.

### NHP is biosynthesized and induces acquired resistance in monocotyledonous plants

**Fig. 5.**
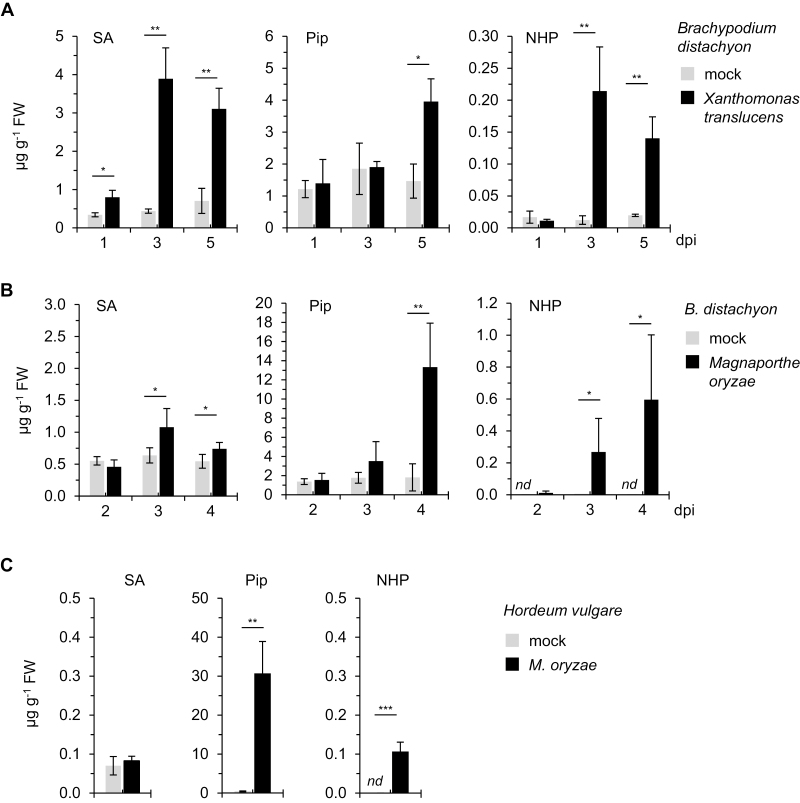
Pathogen-inducible activation of the pipecolate metabolic pathway in response to bacterial and fungal infection in leaves of the monocots *Brachypodium distachyon* and *Hordeum vulgare*. (A) Levels of SA, Pip, and NHP in leaves of *B. distachyon* BD21 inoculated with *Xanthomonas translucens* and in leaves of mock-treated plants. Leaves of 3-week-old plants were infiltrated with a bacterial suspension of OD_600_=0.5 and samples were collected at 1, 3, and 5 dpi (*n*=3). Other details are as described in Fig. 2A. (B) Levels of SA, Pip, and NHP in leaves of *B. distachyon* BD21 inoculated with *Magnaporthe oryzae* strain Guy11 and in leaves of mock-treated plants. Leaves were spray-inoculated with a suspension of fungal spores (50 spores µl^–1^) and samples were collected at 2, 3, and 4 dpi (*n*=3). *nd*, Not detected. (C) Levels of SA, Pip, and NHP in leaves of *H. vulgare* cv. Ingrid inoculated with *M. oryzae* isolate TH6772 and in leaves of mock-treated plants. Leaves were spray-inoculated with a suspension of 250 conidia µl^–1^ and samples were collected at 5 dpi (*n*=3). Data are presented as mean ±SD. Statistically significant differences between samples from pathogen-inoculated and mock-treated leaves at a given time point are indicated with asterisks: ***P<0.001, **P<0.01, *P<0.05 (two-tailed *t*-test).

The Poaceae species *B. distachyon* has been adopted and developed into a useful model for investigating the biology of monocotyledonous plants and cereal crops, including the study of plant immune responses (Fitzgerald *et al.*, 2015; Scholthoff *et al*., 2018). To investigate the occurrence and possible immune function of the NHP pathway in *B. distachyon* BD21, we inoculated leaves of 3-week-old plants with the bacterial leaf streak pathogen *X. translucens* (Fitzgerald *et al.*, 2015). SA started to accumulate from relatively low basal levels in the *B. distachyon* leaves at 1 dpi and reached values of 3–4 µg g^–1^ FW during later stages of infection (3 dpi and 5 dpi; Fig. 5A). These marked increases in the levels of free SA were accompanied by a massive accumulation of SAG and an elevation of the level of SA glucose ester from 3 dpi onwards (Supplementary Fig. S4). The basal levels of Pip in *B. distachyon* leaves, at 1–2 µg g^-1^ FW, were higher than those generally observed for Arabidopsis Col-0 leaves (Návarová *et al.*, 2012), and Pip levels started to accumulate significantly only at later stages of infection (5 dpi), to ~4 µg g^–1^ FW (Fig. 5A). By contrast, NHP levels rose markedly from very low basal levels (<0.03 µg g^–1^ FW) to moderate levels (~0.2 µg g^–1^ FW) at 3 dpi (Fig. 5A).

We also inoculated leaves of *B. distachyon* BD21 plants with the rice blast fungus *M. oryzae*, which has previously been shown to establish compatibility with *B. distachyon* (Parker et al., 2008, 2009). Accumulation of SA in *B. distachyon* leaves was observable in this interaction, but was less pronounced than that in response to *X. translucens* (Fig. 5B). By contrast, the accumulation of Pip was quantitatively higher for the interaction with *M. oryzae* than for that with *X. translucens* at 5 dpi (Fig. 5B). Similarly, NHP accumulated to somewhat greater amounts in the course of *M. oryzae* infection and reached values of ~0.6 µg g^–1^ FW at 5 dpi (Fig. 5B). 

In addition, we analyzed the levels of defense-related metabolites in *Hordeum vulgare* cv. Ingrid inoculated with the compatible *M. oryzae* isolate TH6772 and at a progressed stage of disease (5 dpi; Delventhal *et al.*, 2017). The basal levels of the three metabolites under investigation were low or undetectable in barley leaves, and SA accumulation was not observed after fungal inoculation. However, we detected a strong accumulation of Pip to ~30 µg g^–1^ FW at 5 dpi, and NHP accumulated to moderate but clearly detectable levels (0.1 µg g^– 1^ FW) in *M. oryzae*-infected barley leaves (Fig. 5C).

In summary, our metabolite analyses indicate that monocot plants are able to activate the pipecolate pathway to synthesize both Pip and NHP upon bacterial and fungal inoculation. The SA biosynthetic pathway is induced in parallel, but the degree of induction depended on the particular plant–pathogen interaction.

**Fig. 6.**
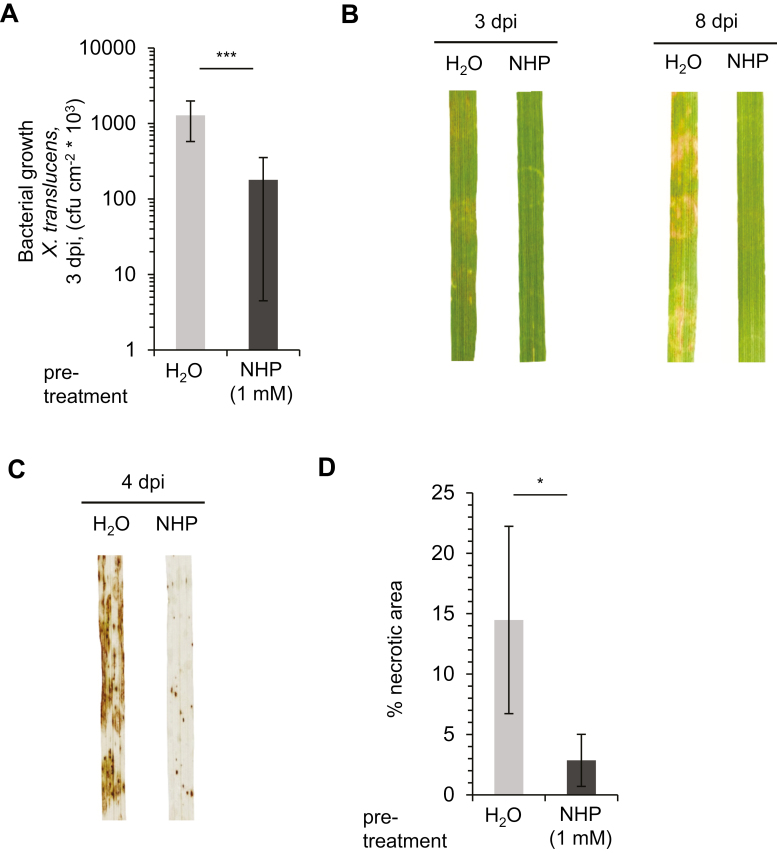
NHP induces acquired resistance to bacterial and fungal infection in *Brachypodium distachyon.* (A) Growth of *Xanthomonas translucens* in leaves of *B. distachyon* BD21. Plants were pretreated by infiltrating 1 mM NHP solution or H_2_O (control) into the leaves. One day later, leaves were pressure-inoculated with a suspension of *X. translucens* of OD_600_=0.5. Bacterial numbers in leaves were assessed at 3 dpi (*n*=10). Other details are as described in Fig. 3A. (B) Representative disease symptoms of *X. translucens*-inoculated *B. distachyon* leaves from plants pretreated with H_2_O or 1 mM NHP at 3 dpi and 8 dpi. (C) Representative destained *B. distachyon* BD21 leaves at 4 dpi after inoculation with *M. oryzae*. Leaves of plants were pretreated with NHP or H_2_O as described in A. One day later, the leaves were spray-inoculated with *M. oryzae* conidia. Brownish areas represent necrotic disease symptoms as a consequence of *M. oryzae* infection. (D) Disease scoring of *B. distachyon* BD21 leaves inoculated with *M. oryzae* after pretreatment with H_2_O or NHP. At 4 dpi, leaves were harvested and destained, and the percentage of necrotic area per leaf surface was determined as a measure of disease severity. Statistically significant differences in bacterial growth (A) or leaf necrotic areas (D) between NHP- and H_2_O-pretreated plants are indicated with asterisks: ***P<0.001, *P<0.05 (two-tailed *t*-test).

To investigate whether NHP also acts as an immune-active substance in monocots, we pretreated the leaves of *B. distachyon* BD21 plants with 1 mM NHP solution or water, inoculated them with *X. translucens* or *M. oryzae* 1 day later, and scored bacterial growth and fungal disease progression in NHP-treated and control plants. With *X. translucens*, the leaves of the control plants hosted ~10-fold higher bacterial numbers than the leaves of NHP-pretreated plants at 3 dpi (Fig. 6A). Moreover, the pronounced brownish necrotic disease symptoms that were evident on the infected leaves of control plants were rarely observed on the leaves of NHP-pretreated plants (Fig. 6B). Following inoculation with suspensions of *M. oryzae* conidia, leaves of *B. distachyon* control plants developed numerous dark brown necrotic lesions at infection sites (Parker *et al.*, 2008). As a measure of disease severity, we assessed the necrotic areas that emerged at 4 dpi on leaf surfaces by quantifying the extent of brownish areas of chlorophyll-destained leaves (Fig. 6C). We observed that pretreatment with NHP significantly attenuated the otherwise widely developing necrotic lesions of *M. oryzae*-inoculated leaves (Fig. 6C, D). Together, these resistance assays indicate that NHP can act as a potent inducer of acquired resistance against leaf infection by both the bacterium *X. translucens* and the fungus *M. oryzae* in the monocot *B. distachyon*.

## Discussion

NHP was identified as a naturally occurring metabolite for the first time in the dicot model species *A. thaliana*. In this plant, NHP accumulated markedly in response to leaf infection by *P. syringae* in both locally inoculated and distal uninfected leaves (Hartmann *et al.*, 2018). Phylogenetic analyses of publicly available genomic and proteomic databases on the basis of *A. thaliana* sequence information suggest a widespread occurrence of homologs of the core NHP biosynthetic pathway genes across the plant kingdom, encompassing monocotyledonous and dicotyledonous plant species alike, many of which are crop species with high commercial or nutritional value (Hartmann *et al.*, 2018; Holmes *et al.*, 2019). In the present study, we show that NHP accumulates in the local and systemic leaves of cucumber upon bacterial inoculation (Fig. 2). Further, NHP biosynthesis and accumulation were observed in the leaves of *P. syringae*-challenged tobacco and tomato, *P. infestans-*infected tomato, *B. distachyon* infected with *X. translucens* and *M. oryzae*, and *M. oryzae*-inoculated barley (Figs 4, 5). In addition to our work, a recent study detected NHP accumulation in tobacco, tomato, and mustard plants when treated with *Pst* DC3000 bacteria, although absolute NHP levels were not determined (Holmes *et al.*, 2019). Therefore, both monocot and dicot plants have the capacity to induce NHP biosynthesis in response to microbial attack. Moreover, leaf infections by diverse bacterial, oomycete, and fungal pathogens can trigger plant NHP biosynthesis.

A common feature of all the species analyzed so far is that NHP biosynthesis is inducible and that the levels of NHP in leaves of unstressed naive plants are very low or, in most cases, not detectable (Hartmann *et al.*, 2018; Figs 2, 4, 5). Thus, in the absence of biotic challenge, plants apparently keep NHP at very low basal levels. Two recent studies have reported that Arabidopsis lines or mutant plants with constitutively elevated levels of NHP show permanently activated defenses and dwarfed phenotypes: specifically, lines overexpressing the calcium-dependent protein kinase CPK5 and mutants in which *CAMTA3*, a negative immune-regulatory gene, is defective (Guerra *et al.*, 2020; Sun *et al.*, 2020). When crossed with the *fmo1* mutant, both lines lose their dwarfism and their ability to synthesize NHP, indicating that the growth retardation is caused by constitutively elevated levels of NHP. This observation suggests that the usually observed low level of the resistance activator NHP in naive plants is a prerequisite for maximal growth performance. The inducible character of NHP biosynthesis would ensure that acquired resistance is switched on only when plants encounter a (biotic) stress stimulus and in this way limit constitutive growth impairment. A time-course analysis in Arabidopsis revealed that the levels of NHP reach a maximum over the course of *P. syringae*-triggered accumulation and later decrease, as would be expected for a signaling molecule (Hartmann *et al.* 2018; Hartmann and Zeier, 2019). Biochemical modification, such as through glycosylation, very likely contributes to the lower levels of free NHP after its stress-induced accumulation (Chen *et al.*, 2018; Hartmann and Zeier, 2018). Accordingly, we detected a stress-inducible NHP-hexose conjugate in Arabidopsis and in cucumber (Hartmann and Zeier, 2018; Supplementary Fig. S1B). However, the levels of this conjugate were below the limit of detection in the other species under investigation.

Pip functions as the direct metabolic precursor of NHP (Hartmann *et al.*, 2018). Previous studies have reported that inoculation with a pathogen markedly induces the accumulation of Pip in different angiosperm species, including Arabidopsis, tobacco, potato, soybean, rice, and barley (Pálfi and Dézsi, 1968; Návarová *et al.*, 2012; Vogel-Adghough *et al.*, 2013; Aliferis *et al.*, 2014; Abeysekara *et al.*, 2016; Ádám *et al.*, 2018; Lenk *et al.*, 2019). Consistently, the results of the present study show that the levels of Pip increase strongly in leaves of tobacco, soybean, and barley inoculated with compatible *Pseudomonas* bacteria (Figs 4, 5). In addition, Pip accumulated to high levels in tomato leaves infected with bacterial and oomycete pathogens (Fig. 4B, C). While most of the species investigated have low basal levels of Pip and show early and strong pathogen-induced accumulation, the situation is different for *C. sativus* and *B. distachyon*. In cucumber, the constitutive levels of Pip were remarkably high (between ~10 and 25 µg^–1^ FW) and did not increase further after *P. syringae* inoculation (Fig. 2A, B). Further, in *B. distachyon*, relatively high basal levels of Pip were detected (1–2 µg^–1^ FW), and the increases in Pip upon pathogen inoculation were slower than the induced accumulation of its derivative NHP (Fig. 5A, B).

Therefore, different mechanisms for the induction of NHP biosynthesis seem to exist in different species. In Arabidopsis, tobacco, tomato, soybean, and barley, NHP accumulates in response to pathogen attack in parallel with its direct metabolic precursor Pip. In addition, the levels of the pathway precursor amino acid lysine increase in these plants (Supplementary Fig. S5; Návarová *et al.*, 2012). The accumulation of Pip in leaf tissue of inoculated Arabidopsis is closely linked to a concomitant increase in the expression of the biosynthetic genes *ALD1* and *SARD4*, and the direct Pip precursor dehydropipecolic acid accumulates to detectable levels (Návarová *et al.*, 2012; Hartmann *et al.*, 2017; Hartmann and Zeier, 2019). The immune-regulatory genes *EDS1* and *PAD4*, as well as several transcription factors, have been shown to regulate inducible *ALD1* gene expression and the biosynthesis of Pip and NHP (Návarová *et al.*, 2012; Hartmann *et al.*, 2018; Sun et al., 2018, 2020; Wang *et al.*, 2018; Kim *et al.*, 2020). Similarly, the pathogen-induced accumulation of Pip in soybean is closely linked to a concomitant transcriptional induction of the soybean *ALD1* homolog *GmALD1* (Abeysekara *et al.*, 2016).

Cucumber belongs to a second category. In this species, high constitutive levels of Pip, associated with constitutive expression of the *C. sativus ALD1* gene, were observed, and the levels of lysine remained unchanged after pathogen inoculation (Fig. 2; Supplementary Figs S2, S5). Although induction of the pathway after *P. syringae* inoculation was associated with further increases in the level of *ALD1* transcripts, the *de novo* expression of *FMO1* after pathogen infection appears to be the major pathogen-inducible trigger that switches on the generation of NHP (Supplementary Fig. S2). In this way, endogenous Pip could be used directly as a substrate as FMO1 accumulates to rapidly induce NHP biosynthesis. The metabolic situation in cucumber corroborates our previous findings that NHP, but not its metabolic precursor Pip, is the actual mediator of resistance, because otherwise, the elevated constitutive levels of Pip would result in constitutive immunity in *C. sativus*. Consistently, Arabidopsis plants defective in the Pip *N*-hydroxylase gene *FMO1* were unable to induce SAR in response to exogenous Pip, but acquired resistance when supplied with exogenous NHP (Hartmann *et al.*, 2018). The situation in *B. distachyon* appears to be intermediate: although the whole metabolic pathway is induced upon pathogen attack, an early accumulation of NHP might be favored by the relatively high constitutive levels of the Pip precursor (Fig. 5A, B; Supplementary Fig. S2).

Our present and previous results show that NHP is able to induce acquired resistance in distinct angiosperm species. On the one hand, genetic and biochemical evidence demonstrated that the endogenous accumulation of NHP is required for pathogen-induced SAR in Arabidopsis. On the other hand, exogenously applied NHP induced SAR to *P. syringae* and *H. arabidopsidis* infection in Arabidopsis and restored the defects in acquired resistance of the NHP-deficient *ald1* and *fmo1* mutants (Hartmann *et al.*, 2018; Chen *et al.*, 2018). Although the cellular compartment(s) in which NHP acts to trigger SAR and what concentration of NHP is necessary for biological activity in target cells remain elusive, our feeding experiments indicate that effective doses of exogenously applied NHP per plant below 1 µmol are sufficient to activate SAR (Fig. 1). In addition to our results, Holmes *et al.* (2019) recently reported that exogenously applied NHP induces SAR in tomato and pepper (*Capsicum annuum*) to *Xanthomonas euvesicatoria* and *Pst* DC3000 infection, respectively. Here, we show that *C. sativus* and tobacco plants exogenously treated with NHP acquire resistance to *P. syringae* infection (Fig. 3). In addition to these dicots, the monocot *B. distachyon* acquires resistance to both *X. translucens* and *M. oryzae* infection when supplied with NHP (Fig. 6). Together, these findings indicate that NHP functions as a general and conserved regulator of SAR in angiosperms. NHP mediates the resistance of both mono- and dicotyledonous plant species against infection with bacterial, oomycete, and fungal pathogens. Such an activation of broad-spectrum plant resistance has long been considered a hallmark of SAR (Sticher *et al.*, 1997).

Plants with activated SAR are primed for a fortified induction of defenses when attacked by pathogens (Jung *et al.*, 2009; Návarová *et al.*, 2012). Defense priming in Arabidopsis is completely absent in the NHP-deficient *ald1* and *fmo1* mutants, indicating a central role for NHP in this process (Návarová *et al.*, 2012; Bernsdorff *et al.*, 2016). NHP-induced resistance and SAR-related defense priming are substantially diminished in the SA-deficient *sid2* mutant (Bernsdorff *et al.*, 2016; Hartmann *et al.*, 2018). Moreover, Pip primed plants for enhanced SA-induced expression of *PR* genes in an *FMO1*-dependent manner (Bernsdorff *et al.*, 2016). These observations suggest a close, coordinated interplay between NHP and SA for the effective activation of SAR and defense priming (Hartmann and Zeier, 2019). Our metabolite analyses show that in the leaves of pathogen-inoculated Arabidopsis, cucumber, tobacco, tomato, soybean, and *B. distachyon*, SA (and, commonly, SA derivatives such as SAG) accumulates in parallel with NHP, so that, in principle, such a positive interplay for effective SAR induction is possible in these species.

Exceptionally, although barley leaves accumulated Pip and NHP upon *M. oryzae* infection, SA levels were low and did not change upon inoculation (Fig. 5C). Similarly, SA did not accumulate in *H. vulgare* infected with the powdery mildew pathogen *Blumeria graminis* f. sp. *hordei* (Hückelhoven *et al.*, 1999). Therefore, SA might not function as a signaling molecule for SAR in barley. In line with this assumption, a previous study has found that *H. vulgare* does not show enhanced pathogen resistance in response to exogenous SA treatments (Dey *et al.*, 2014), which is a common feature in many other species. However, barley plants can acquire resistance upon treatment with the synthetic resistance activator 2,6-dichloroisonicotinic acid (Kogel *et al.*, 1994). Our metabolite analyses suggest that an endogenous activation of SAR in barley might proceed via NHP signaling. This assumption is compatible with the finding that *H. vulgare* plants fed with exogenous Pip acquired resistance to *X. translucens* infection (Lenk *et al.*, 2019).

The molecular mechanisms for SAR in monocots are not as well understood as those in dicots (Shah and Zeier, 2013; Balmer *et al.*, 2013). Our findings that *B. distachyon* shows accumulation of Pip and NHP upon inoculation with both *X. translucens* and *M. oryzae*, and that NHP treatment induces acquired resistance in *B. distachyon* to infection by the same pathogens, strongly suggest that NHP functions as a central player in SAR in this species (Figs 5, 6). The capacities established for *B. distachyon* as a model monocot hold the promise of further investigations of this aspect at the genetic level (Fitzgerald *et al.*, 2015; Scholthoff *et al*., 2018). Overexpression of the first NHP pathway gene, *ALD1*, in rice also enhanced resistance to infection by *M. oryzae* (Jung *et al.*, 2016), which might indicate a function of NHP for SAR in rice as well. Together, the existing data on *B. distachyon*, barley, and rice suggest that activation of SAR by NHP might be a conserved feature of monocotyledonous plants. Our metabolic data further show that SA strongly accumulates in *B. distachyon* leaves upon *X. translucens* infection, while SA levels were only moderately increased in response to *M. oryzae* inoculation (Fig. 5). Therefore, SA might well contribute to SAR in *B. distachyon*. In this light, a general separation of the molecular bases of SAR into “monocot” and “dicot” mechanisms might not be helpful. Rather, the degree of induction of different SAR-activating pathways appears to vary within distinct species and depends on the nature of the attacking pathogen.

An interesting—and controversial—mechanistic aspect of SAR is how plants achieve long-distance communication between inoculated and distant leaves (Shah and Zeier, 2013). When NHP was locally applied to individual rosette leaves of Arabidopsis by infiltration, it induced defense-related gene expression and a strong SAR response in distant leaves (Fig. 1; Chen *et al.*, 2018). Similarly, local application of NHP to leaflets proximal to the stem induced systemic resistance in distal leaflets of the same tomato leaf (Holmes *et al.*, 2019). Moreover, transient *Agrobacterium*-mediated expression of the Arabidopsis NHP biosynthetic genes *ALD1*, *SARD4*, and *FMO1* in *Nicotiana benthamiana* led to an accumulation of NHP in the absence of pathogen inoculation. When the same set of genes was transiently expressed in proximal leaflets of tomato leaves, systemic immunity was induced in distal leaflets (Holmes *et al.*, 2019). These results support a possible function of NHP as a mobile SAR signal, but the experimental setups do not clarify whether the NHP that accumulates biologically during a SAR-inducing plant–pathogen interaction in inoculated leaves ultimately travels to distant leaves and induces SAR. On the basis of these results, it is also possible that NHP triggers a long-distance communication process in the inoculated tissue that is based on cell-to-cell signal propagation.

Previous efforts to identify SAR-related mobile signaling compounds investigated the changes in the phloem sap of cucumber plants following SAR induction by pathogen infection. Upon inoculation of the first leaf of cucumber with necrotizing pathogens, Métraux *et al.* (1990) observed increases in the levels of SA in collected phloem sap that preceded the induction of resistance in the second (systemic) leaf. Studies using putative radiolabeled biosynthetic precursors of SA concluded that SA is transportable from lower inoculated to upper cucumber leaves, but explicitly pointed out the potential existence of a different primary signal that induces *de novo* synthesis of SA in upper leaves (Meuwly *et al.*, 1995). In line with the latter suggestion, systemic defense responses in distal cucumber leaves were observed before increases in SA levels were detectable in the phloem sap of inoculated leaves (Rasmussen *et al.*, 1991). In addition to SA, its structural isomer, 4-hydroxybenzoic acid, was found to accumulate in the phloem sap from inoculated leaves of cucumber, but its relation to SAR remained unclear (Smith-Becker *et al.*, 1998). Using *Psl* inoculation of the first leaf of cucumber, we confirmed that SA and its derivative, SAG, strongly accumulate in the inoculated leaves and the phloem sap of these leaves, but only SAG accumulated substantially in the exudates of systemic leaves (Fig. 2; Supplementary Fig. S1). By contrast, the levels of NHP increased strongly in the inoculated leaves and their phloem sap, in the phloem sap of distant leaves and, finally, in the distant leaf tissue itself (Fig. 2). These findings support the hypothesis that NHP might function as a mobile primary inducer of SAR within a biological plant–pathogen interaction. Its biosynthetic precursor Pip, which was previously identified as a constituent of the phloem in cucumber (Hu *et al.*, 2016), occurs at very high constitutive levels in both cucumber leaves and exudates and does not increase in response to the pathogen stimulus (Fig. 2). This observation argues against a function of Pip as a mobile SAR signal, but indicates that Pip could be a ready-to-use precursor in cucumber leaf and phloem tissue that is converted into the primary, and presumably phloem-mobile, SAR inducer NHP in response to pathogen attack.

## Conclusions

The current study shows that the biosynthesis of NHP is induced in diverse monocot and dicot plants species upon inoculation with compatible pathogens. In addition, NHP functions as a potent activator of acquired resistance in both mono- and dicotyledonous plants. The systemic accumulation of NHP in leaves and phloem sap of inoculated cucumber plants is consistent with a possible function of NHP as a primary, mobile SAR-inducer.

## Supplementary data

Supplementary data are available at *JXB* online.

Fig. S1. Levels of glycosylated salicylic acid and *N*-hydroxypipecolic acid conjugates in leaves and phloem sap of *Cucumis sativus*.

Fig. S2. Transcript levels of *Cucumis sativus ALD1* and *FMO1* in *P. syringae* pv. *lachrymans*-inoculated and mock-treated leaves.

Fig. S3. Exogenous application of Pip and NHP elevate resistance of tobacco to *P. syringae* pv. *tabaci* infection.

Fig. S4. Levels of SA-β-glucoside and SA glucose ester in leaves of *Brachypodium distachyon*.

Fig. S5. Levels of lysine in pathogen-inoculated leaves of different plant species.

Table S1. Plant growth conditions.

Table S2. Primers and conditions used for RT–PCR analysis.

eraa317_suppl_Supplementary_Figure_and_Table
