# Supplementary material for: Inducible biosynthesis and immune function of the systemic acquired resistance inducer N-hydroxypipecolic acid in monocotyledonous and dicotyledonous plants
Source: J Exp Bot. 2020 Jul 6;71(20):6444–59. doi: 10.1093/jxb/eraa317 (PMC7586749; doi:10.1093/jxb/eraa317)
Supplement: eraa317_suppl_Supplementary_Figure_and_Table [file eraa317_suppl_supplementary_figure_and_table.pdf]

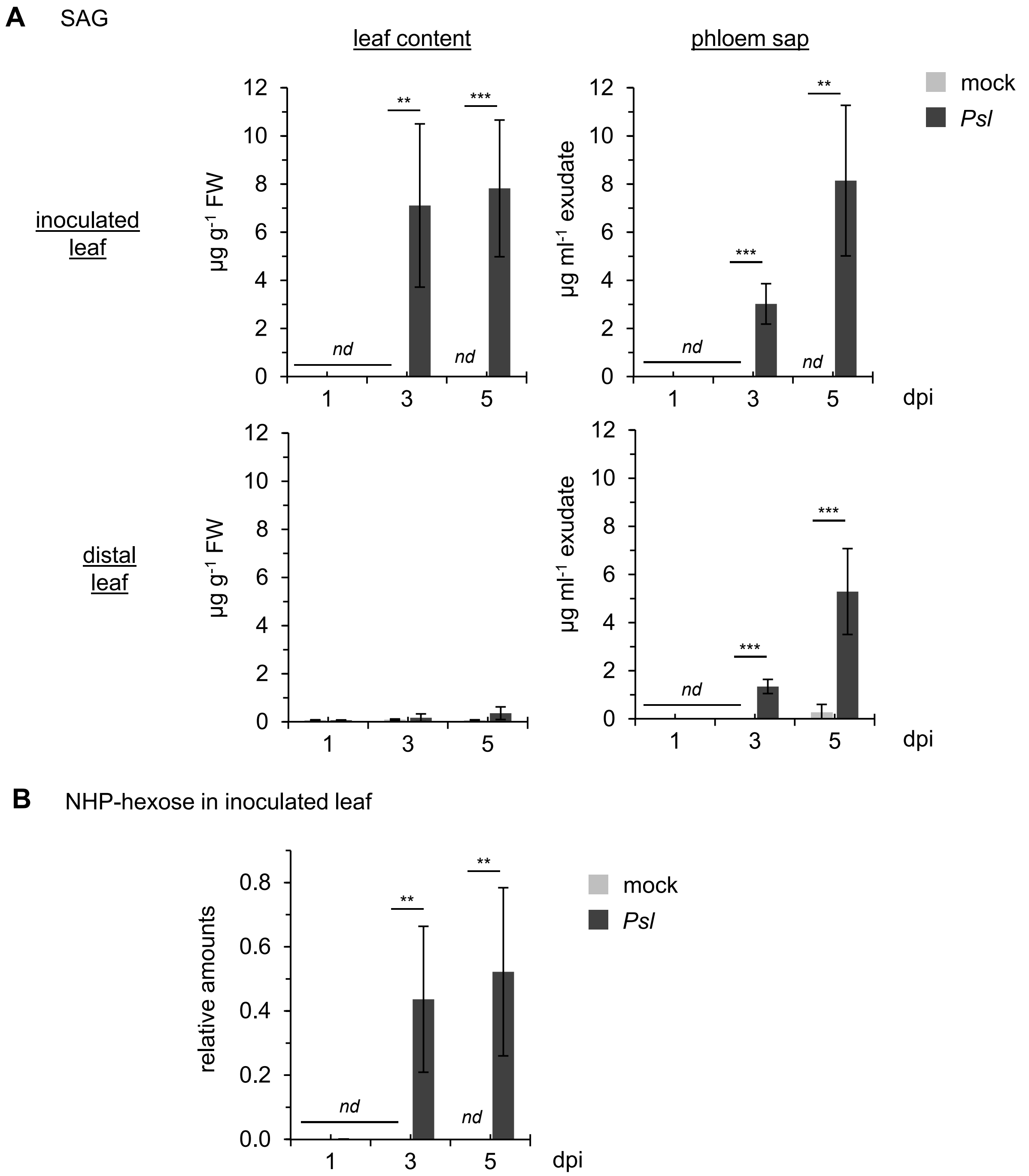

Fig. S1. Levels of glycosylated salicylic acid (SA)- and N-hydroxypipecolic acid (NHP)-conjugates in leaves and phloem sap of *Cucumis sativus*.

A, Levels of salicylic acid-β-glucoside (SAG) in *C. sativus* leaves and phloem sap after mock-treatment and *P. syringae* pv. *lachrymans* (*Psl*)-inoculation. Top left: inoculated leaves. Bottom left: distal (systemic) leaves. Top right: phloem sap from inoculated leaves. Bottom right: phloem sap from distal leaves. Samples were collected at 1, 3, or 5 days post inoculation (dpi). Statistically significant differences between *Psl*- and mock-control samples are indicated with asterisks (***: $P < 0.001$, **: $P < 0.01$; two-tailed *t* test). *nd* = not detected. Other details as described in Fig. 1.

B, Levels of a conjugated NHP-hexose derivative (Hartmann and Zeier, 2018) in inoculated *C. sativus* leaves. Relative amounts are given. The compound was neither detected in distal leaves nor in leaf phloem sap.

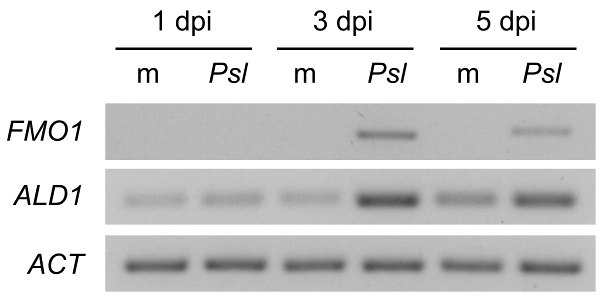

Fig. S2. Transcript levels of *Cucumis sativus ALD1* and *FMO1* in *P. syringae* pv. *lachrymans*-inoculated and mock-treated leaves.

Gene expression was assessed by RT-PCR analysis. See Fig. 1A and Table S2 for experimental details. m: mock-treated leaves; *Psl: P. syringae* pv. *lachrymans*-inoculated leaves; dpi: days post inoculation.

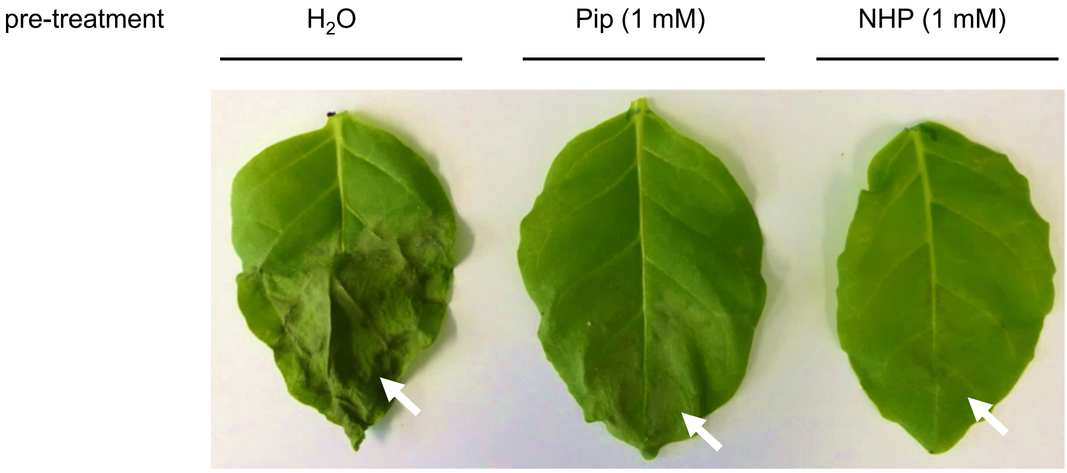

Fig. S3. Exogenous application of Pip and NHP elevate resistance of tobacco to *P. syringae* pv. *tabaci* infection.

Disease symptoms (3 dpi) of leaves of *Nicotiana tabacum* cv. Xanthi from plants pre-treated with 10 ml of H_2O, 1 mM Pip, or 1 mM NHP and inoculated with *P. syringae* pv. *tabaci* (OD_{600} = 0.005) one day later. White arrows indicate inoculated leaf areas.

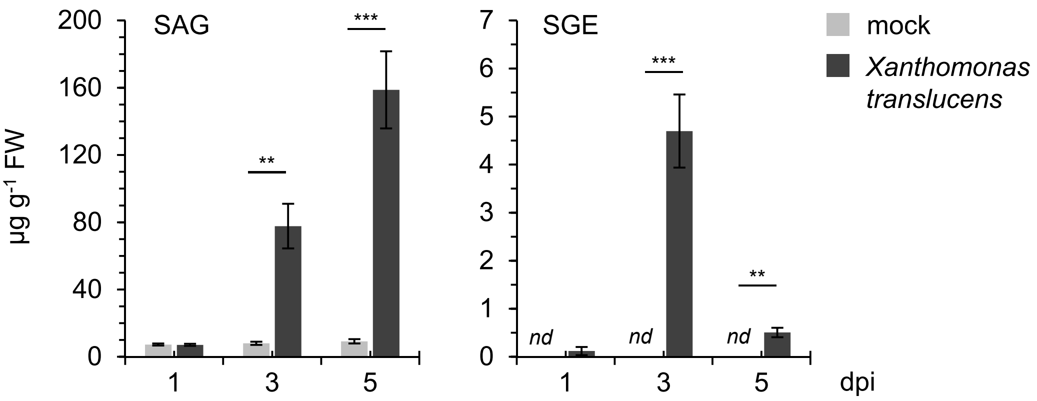

Fig. S4. Levels of SA-β-glucoside (SAG) and SA glucoseester (SGE) in leaves of *Brachypodium distachyon.* Levels of SAG (left) and SGE (right) in leaves of *B. distachyon* BD21 inoculated with *Xanthomonas translucens* and in leaves of mock-treated plants. Samples were collected at 1, 3, or 5 days post inoculation (dpi). *nd* = not detected. Other details as described in Fig. 5A.

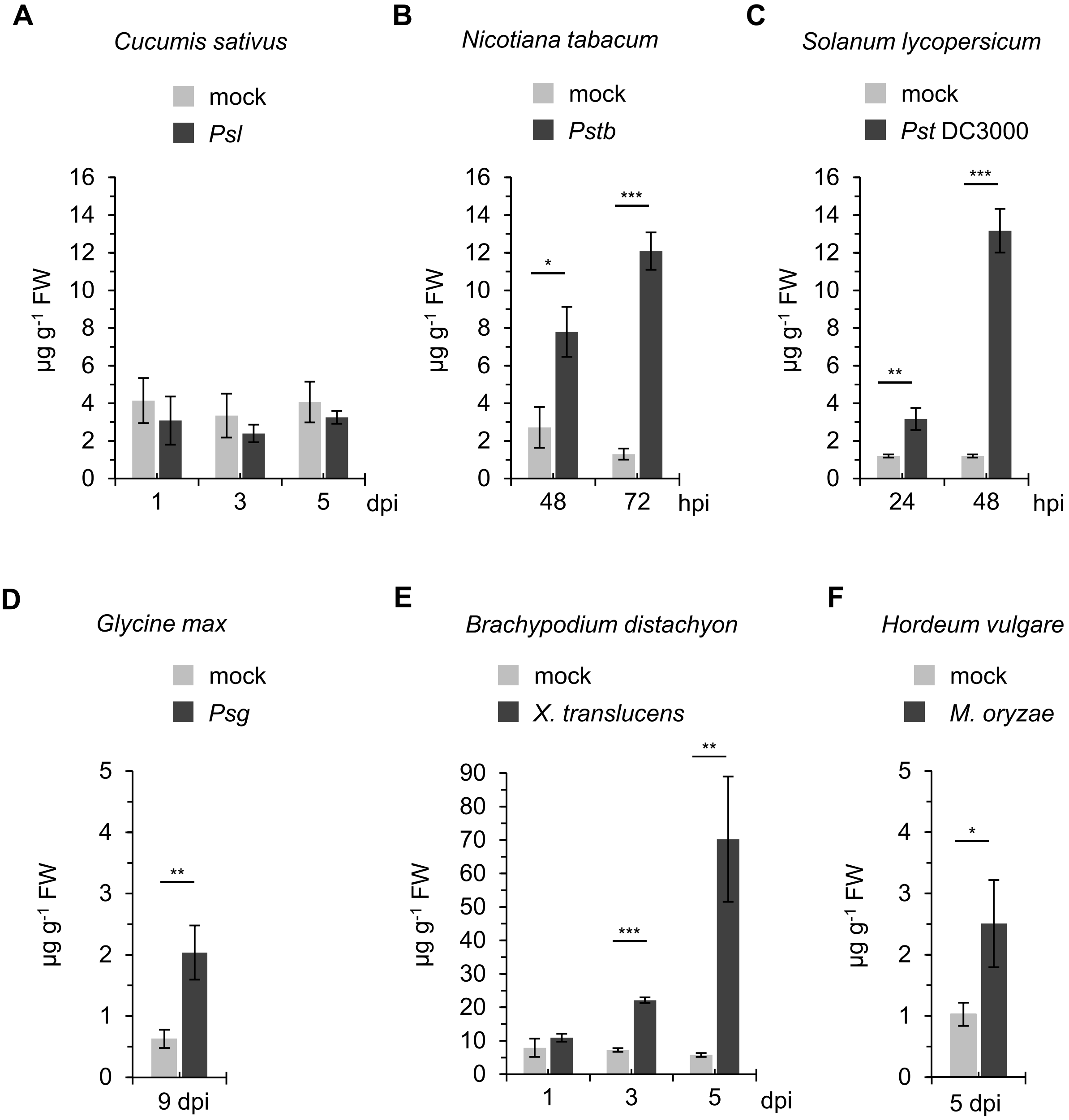

Fig. S5. Levels of lysine in pathogen-inoculated leaves of different plant species.

A, Leaves of *Cucumis sativus* cv. Wisconsin SMR58 inoculated with *Pseudomonas syringae* pv. *lachrymans* (*Psl*) . See Fig. 2A.

B, Leaves of *Nicotiana tabacum* cv. Xanthi with *P. syringae* pv. *tabaci* (*Pstb*) . See Fig. 4A.

C, Leaves of *Solanum lycopersicum* cv. Heinz1706 with *P. syringae* pv. *tomato* (*Pst*) DC3000 . See Fig. 4B.

D, Leaves of *Glycine max* cv. Maple Arrow with *P. savanastoi pv. glycinea* (*Psg*) . See Fig. 4D.

E, Leaves of *Brachypodium distachyon* BD21 with *Xanthomonas translucens* . See Fig. 5A.

F, Leaves of *Hordeum vulgare* cv. Ingrid with *Magnaporthe oryzae* . See Fig. 5C.

Statistically significant differences between pathogen- and mock-samples are indicated with asterisks (***: $P < 0.001$, **: $P < 0.01$; two-tailed *t* test).

Table S1. Plant growth conditions.

**Table S1.** Plant growth conditions. **Plant material**		**Growth condtions**			
**Plant species**	**Cultivar**	**Pre-treatment / germination**	**Growth medium**	**Light (°C) / dark (°C)-cycle**	**Relative humidity**
Cucumis sativus	Wisconsin SMR58	-	soil[1]/vermiculite/sand (8:1:1)	16 h (25 °C) / 8 h (23 °C)	60%
Nicotiana tabacum	Xanthi	-	soil[1]/vermiculite/sand (8:1:1)	16 h (23 °C) / 8 h (20 °C)	60%
Solanum lycopersicum	Heinz1706	-	soil[1]/vermiculite/sand (8:1:1)	16 h (23 °C) / 8 h (20 °C)	60%
Glycine max	Maple Arrow	-	soil[1]/vermiculite/sand (8:1:1)	16 h (25 °C) / 8 h (23 °C)	70%
Brachypodium distachyon	Bd21	surface sterilization, incubation on half-MS agar plates for 3 days (darkness, 4°C), then transfer	soil[1]/sand/vermiculite/gravel (4:2:1:1)	16 h (25 °C) / 8 h (23 °C)	60%
Hordeum vulgare	Ingrid	pre-germination for 24 h on wet filter paper, then transfer	standard soil (type ED73)	16 h (20 °C) / 8 h (20 °C)	60%

[1]Substrat BP3; Klasmann-Deilmann

Table S2. Primers and conditions used for RT-PCR analysis.

**Gene (Transcript Accession)**	**Primer Sequence (5‘‘)**	**Annealing temperature (°C)**	**PCR cycles**
Actin (XM_004147305.2)	Forward: GCATCGCTCAGTACCTTCCA<br>Reverse: GACCCGTATCTGAGCTCAACC	63	35
FMO1 (XM_004144232.1)	Forward: GACTATGCGAAGCTCGACCA<br>Reverse: AGGAGGCCTTGATTAGGGGT	62	38
ALD1 (XM_011651615.1)	Forward: CCAAATTCGCTGGCTTCACC<br>Reverse: TGGAAAGGCAAGCAAGACCA	61	33
